# Fingers Crossed! An Investigation of Somatotopic Representations Using Spatial Directional Judgements

**DOI:** 10.1371/journal.pone.0045408

**Published:** 2012-09-24

**Authors:** Alyanne M. de Haan, Helen A. Anema, H. Chris Dijkerman

**Affiliations:** 1 Experimental Psychology, Helmholtz Institute, Utrecht University, Utrecht, The Netherlands; 2 Department of Public Health, Academic Medical Centre, University of Amsterdam, Amsterdam, The Netherlands; University of Reading, United Kingdom

## Abstract

Processing of tactile stimuli requires both localising the stimuli on the body surface and combining this information with a representation of the current posture. When tactile stimuli are applied to crossed hands, the system first assumes a prototypical (e.g. uncrossed) positioning of the limbs. Remapping to include the crossed posture occurs within about 300 ms. Since fingers have been suggested to be represented in a mainly somatotopic reference frame we were interested in how the processing of tactile stimuli applied to the fingers would be affected by an unusual posture of the fingers. We asked participants to report the direction of movement of two tactile stimuli, applied successively to the crossed or uncrossed index and middle fingers of one hand at different inter-stimulus intervals (15 to 700 ms). Participants almost consistently reported perceiving the stimulus direction as opposite to what it was in the fingers crossed condition, even with SOAs of 700 ms, suggesting that on average they did not incorporate the unusual relative finger positions. Therefore our results are in agreement with the idea that, by default, the processing of tactile stimuli assumes a prototypical positioning of body parts. However, in contrast to what is generally found with tactile perception with crossed hands, performance did not improve with SOAs as long as 700 ms. This suggests that the localization of stimuli in a somatotopic reference and the integration of this representation with postural information are two separate processes that apply differently to the hands and fingers.

## Introduction

When you want to respond to a tactile stimulus you first have to find out where on the skin this stimulus is located. Moreover, you need to know the location of that skin in space. Thus, in order to locate a tactile stimulus in external space one has to generate a representation of the stimulus position on the skin surface and since your body parts move freely in space, information about the current posture needs to be additionally integrated [1].

Body part posture affects somatosensory processing [2], [3], particularly when the posture is not commonly used during haptic exploration. Yamamoto & Kitazawa for instance [4] (see for similar results [5]–[9]), used a temporal order experiment to investigate how such an unusual body part position affects tactile information processing. It was shown that when tactile stimuli were subsequently applied to crossed hands, a position in which the external spatial frame is incongruent with the somatotopic frame [6], [10], participants tended to misperceive the order in which the tactile information was applied, but only when stimuli were administered in rapid succession. This decline in performance with the hands crossed over the midline is often called the “crossing effect”. The authors concluded that tactile information was first processed according to a prototypical positioning of the limbs and that the second tactile stimulus was applied before the external reference frame was integrated in the representation of the first stimulus. This resulted in confusion of the temporal order of tactile stimuli to crossed hands to such an extent that a quarter of the participants reported a complete reversal of the perceived order of stimuli with stimulus onset asynchronies (SOAs) of up to 300 ms. With larger SOAs, the reversal errors diminished to an average percentage of 5% (95% correct). Similarly, saccades to tactile stimuli on crossed hands tend to be directed to the unstimulated hand at first, but bent to the opposite side after about 200–300 ms ([11]: range 248–319 ms, [12]: a time estimate is not reported, but inspection of their figure 6F would suggest a range between 200–300 ms). In contrast, using a crossmodal cueing paradigm, Azañón et al. [13] concluded that remapping would already be completed somewhere between 180 to 360 ms after stimulus onset. In their supplementary data they investigated the time course of their tactile-visual cueing effects and added intervals of 10, 90 and 140 ms. The data showed that with a 10 ms stimulus interval, no cueing effects were yet shown. With a 30 or 60 ms stimulus interval, cueing effects were based on a somatotopic reference frame, and at a 360 ms interval on a spatiotopic reference frame. With 90, 140 and 180 ms intervals, no significant cueing effect was found, reflecting a time interval in which none of the possible reference frames is dominant, probably due to an incomplete remapping process. In line with this result, using ERPs Heed & Röder [14] found an indication of the use of both an anatomical and a spatial reference frame 100–140 ms after a tactile stimulus on the hand. Thus, crossing effects are often so large that on average a reversal of response patterns is observed. This “reversal effect” in hands (and feet, see [15]) seems quite robust for short stimulus intervals, although time estimates of this phase differ, ranging from around 150 to 300 ms (see for instance [4], [11]–[14]). Longer stimulus intervals provide the opportunity to incorporate the unusual postures into the spatial representation of the tactile stimulus, albeit not completely (on average 95% correct) [5]–[7], [9], [12].

An important question is whether similar patterns of spatial mislocalisation of tactile stimuli for unusual postures can also be found for the fingers, as several others have suggested that somatic finger representations may be somewhat different to hand and arm representation [16]–[19]. There is some evidence suggestive of a misperception of tactile stimuli when applied to crossed fingers of one hand. The Aristotle illusion, for instance, entails that when a small sphere such as a marble is touched with crossed fingers, it is perceived as being two separate marbles. A possible explanation might be that simultaneously applied tactile stimuli at two skin regions that are not normally adjacent are processed as originating from different objects. Benedetti further explored this illusion and performed several studies of tactile perception or haptic exploration with crossed fingers in the 1980s. He showed that when having to report the location of two tactile stimuli applied to (unseen) crossed fingers of one hand, the locations of the stimuli were perceived as if the fingers were uncrossed [20], in contrast to when the spatial position of the fingers with respect to each other was reported [21]. Furthermore, this illusory tactile reversal influenced goal directed movement to tactile targets, and while feedback and training allowed participants to rapidly change their motor behaviour, the stimuli were still perceived as reversed [22]. In line with Yamamoto and Kitazawa's interpretation of their results, it seems that these simultaneously applied tactile stimuli were processed according to a prototypical, uncrossed position of the limbs. Only after several months of training tactile perception with crossed fingers, by fixating the fingers in a crossed position for several hours per day, months in a row, subjects were able to integrate the crossed posture [23]. In contrast to Yamamoto and Kitazawa [4], Benedetti did not vary the stimulus onset asynchrony between the two tactile stimuli, as they were applied simultaneously. The question therefore remains whether the decay of the ‘reversal effect’ (spatial reversal in response pattern) observed in hands is similar for crossed fingers and participants incorporate the crossed finger posture with longer SOAs.

Over the last decade various studies have been published investigating perception of successive tactile stimuli applied to crossed fingers of one hand using several SOAs [24]–[26]. Craig [24] for instance used moving tactile patterns applied to crossed or uncrossed fingers. The two moving patterns were applied in succession to each finger (SOA range = 13–400 ms) and participants had to indicate which of two fingers was stimulated first; left or right (temporal order judgement). Stimulating two fingers in succession induced a perception of motion. Therefore, the direction of motion evoked by the tactile patterns could be either congruent or incongruent with that of the apparent motion evoked by the successive stimuli on the two fingertips. They observed an overall advantage for congruent stimuli that disappeared when SOAs were longer than (about) 200 ms, so the direction of the local motion influenced the order judgement at shorter SOAs. Interestingly, this influence did not differ between the fingers crossed and fingers uncrossed condition. Therefore, it was concluded that, contrary to Benedetti's findings, the crossed finger position was integrated in the processing of the tactile stimuli.

Sekine and Mogi [25] investigated the processing of two tactile stimuli successively applied to crossed fingers using only one SOA of 500 ms. Participants were required to judge which finger received the second stimulus (index or middle finger), or had to judge the direction of stimulation (leftward or rightward). They observed that the TOJ judgments (finger identity) were performed accurately (about 96%) whereas the directional judgments were significantly less accurate, particularly in palm down posture (only 41% correct). The authors concluded that judging which body part has been touched and localising stimuli in external space are distinct processes, with the latter failing to include the crossed finger posture.

Using a series of experiments Heed et al. [26] specifically questioned whether finger position is remapped into external space, hence an incorporation of the crossed posture, and tested this with various SOAs (20 ms–1000 ms) and a temporal order judgment task (middle finger or ring finger first). In contrast to Sekine and Mogi [25], who did not vary the SOA, Heed et al. [25] showed that the proportion of accurate TOJs declined considerably (to an almost flat -but not reversed- psychometric curve at about 60% correct) when the fingers were crossed. They concluded that the response pattern suggests an external coding of the tactile stimuli, indicating that the crossed posture was incorporated in the processing. However, large individual differences in the proportion of accurate TOJs as a function of SOA played a considerable role in producing the on average large crossing effect (e.g. the substantial drop of performance with crossed fingers). It could be that some participants showed a large reversal effect and others did so to a lesser extent, in line with the observations of Yamamoto and Kitazawa [4].

In all, it remains to be investigated at what SOA the “reversal effect” in fingers that is observed in Benedetti's experiments, decays. That is, the time course of the integration of crossed postures when processing tactile stimuli to the fingers still needs to be assessed. To that purpose we used a directional spatial judgement task of tactile stimuli presented to the (unseen) index and middle finger of one hand in a fingers-crossed and uncrossed condition. To explore the temporal aspects of the process, the tactile stimuli were applied with different inter-stimulus intervals ranging from 15 to 700 ms.

## Materials and Methods

### Participants

10 undergraduate, graduate and PhD students of Utrecht University (mean age 23.2 years SD 2.9) participated in this study and received either a small payment or course credits as compensation for their time. All were right-handed, female and had normal tactile sensitivity by self-report. They were naïve to the purpose of the study and gave their informed consent prior to the experiment. The experiment was conducted in agreement with the local ethics and safety guidelines, which are based on the Declaration of Helsinki.

### Apparatus and stimuli

Tactile stimuli consisted of metallic pins with a diameter of 2 mm and were applied using computer controlled miniature solenoid tappers (MSTC3 M&E Solve, Rochester, UK). The tappers were attached to the ventral side of the middle and index fingertip of the left hand with medical tape. All taps had a duration of 6 ms and were given with an intensity that was reportedly well above threshold (at 20% intensity) but not discomforting. Since the tappers produce noise during stimulus presentation, participants wore noise-cancelling headphones during the experiment.

**Figure 1:**
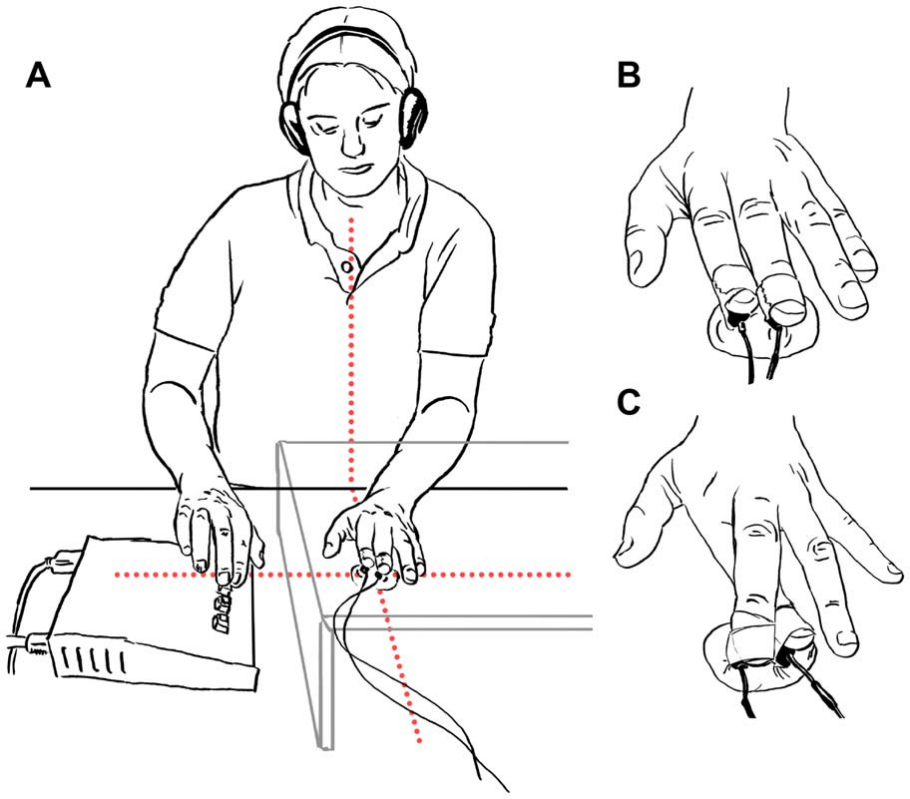
The experimental setup. Participants were seated at a table with their left hand palm down in front of them. The stimulated left hand is occluded from vision by a hardboard cover, on which a red fixation cross was present directly above the stimulated fingers. The right hand is used for responses. The light dotted lines indicate that the hands were placed next to each other, while the stimulated fingers and the gaze were aligned with the body midline (A). Panel B and C show the uncrossed and crossed positioning of the fingers.

Participants were seated at a table in a dimly lit room with their left hand palm down in front of them on the body midline. All tactile stimuli were presented to the left hand, which was occluded from view by a hardboard cover (see figure 1A). The perceived direction in space was recorded by means of a response box. Participants were asked to fixate on a red dot, painted on the hardboard cover above the left hand (see figure 1A).

The middle and index finger of the left hand were either crossed (middle over index) or uncrossed within a block of trials. Clay molds were used to ensure that the distance between the fingertips remained equal (2 cm) between conditions and throughout the experiment (see figure 1B and C). The clay molds did not fixate the fingers in a way that would allow relaxation of the muscles, so the finger crossing had to be maintained actively during the experiment.

### Procedure

Within a trial, two tactile stimuli (taps) were applied successively at one of seven different stimulus onset asynchronies (SOA) (15, 30, 60, 90, 150, 400 & 700 ms) and two directions (leftwards and rightwards). Trials were divided in eight blocks of 56 trials, participants had their fingers crossed in four blocks and uncrossed in the other four. The blocks were presented in a pseudo-randomised order. In all, each finger positioning (2)×direction (2)×SOA (7) condition was tested 16 times. Stimulus onset asynchrony and direction of taps were randomised within blocks, and to ensure that participants remained focused on the task the inter-trial interval was variable (between 1000 and 3000 ms).

Participants were instructed to report the perceived direction in space by pressing one of two buttons on the response box with both the middle and index fingers of the right hand (top button for “leftwards” and bottom for “rightwards”, or vice versa for half of the participants). In-between trials both fingers rested on a centre button of the response box, which was located between the two response keys.

Before the experiment, participants were given instructions about the task and 20 practice trials (10 with uncrossed, 10 with crossed fingers).

### Data analysis

Trials with reaction times longer than 3000 ms (0.76%) and trials in which a response was given before the second tap (0.11%) were excluded from further analysis. In two participants, one block of trials had to be excluded due to technical problems or a failure to follow the instructions.

Both the directional judgements and the reaction times (with respect to the second tap) were recorded and analysed per stimulus onset asynchrony for crossed and uncrossed fingers. We analysed the proportion of rightward directional judgements per SOA. This measure reflects the error rate, but also the influence of SOA on this error rate, thus providing insight into the time course of the effects. Therefore, we consider this analysis to be the most relevant for the influence of SOA. If the crossed posture is correctly integrated during the processing of tactile stimuli to the fingers, crossing the fingers will not interfere with the task and directional judgements with crossed and uncrossed fingers should be the same. However, if directional judgements with crossed fingers are generally based on a somatotopic representation that is not integrated with postural information, we would expect to see an on average inverted relation between SOA and responses to the tactile stimuli on crossed fingers as compared to uncrossed fingers. To investigate this, a logistic function fitting algorithm (Ezyfit Matlab toolbox, logistic model:
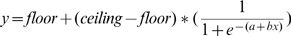
where ‘y’ is the probability of a response rightwards, ‘a’ represents the slope and ‘b’ the point of subjective equality, e.g. the SOA at which a participant responses were 50% rightwards) was applied to both the average group results and the single subject data (see for instance [27] or [28] for a similar logistic model fitting). We used the slope of the fitted function (‘a’ in the model) as a measure of interference of the crossed posture with the directional judgement: if the crossed posture is not correctly integrated, the slope parameters in crossed and uncrossed condition are expected to differ. Cadieux et al. [7] proposed an alternative measure that compares the difference in the total percentage of correct responses over all SOAs between crossed and uncrossed condition. This gives a measure of the average performance with crossed or uncrossed fingers. We included this analysis (adapted in line with Heed et al. [26] for use in a within subjects design) to increase the comparability of this study with other experiments.

The slope parameters and absolute slope parameters (e.g. the numerical values without regards to their signs) of the functions (‘a’ in the model) with crossed and uncrossed fingers for each participant were compared with a Paired Sample T-test. The latter does not give direct additional information about the reversal effect, but gave the possibility to check for differences in general performance (a steeper slope reflects a better performance [15], [26], [27]). Since slower responses are generally found with crossed fingers (or hands), median reaction times were analysed with a finger position (2)×SOA (7) repeated measures ANOVA.

## Results

**Figure 2:**
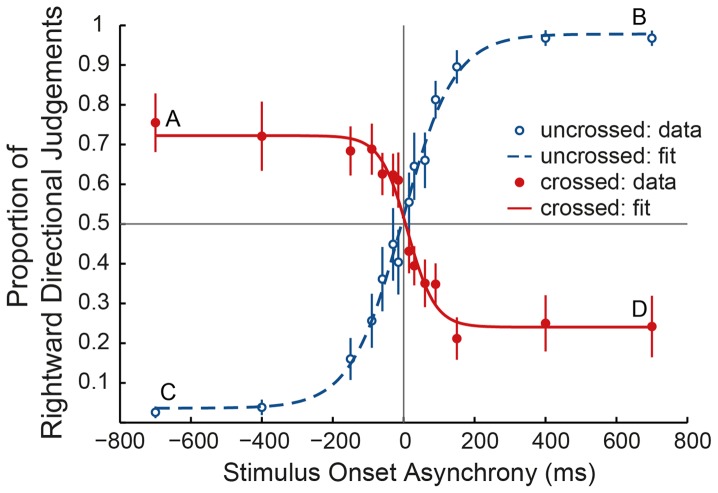
The proportion of rightward directional judgements at the different SOAs. The average proportion of trials in which participants reported the direction of the stimuli to be to the right (in space) is depicted in the fingers uncrossed (blue, open circles) and crossed (red, filled circles) condition at different SOAs, together with the corresponding fitted logistic curves (red continuous line for crossed, blue dashed line for uncrossed condition). Negative SOAs indicate conditions in which the direction of stimuli was to the left in space. So, data points in the bottom left and top right quadrant represent conditions in which on average on more than 50% of the trials a correct response was given. Error bars indicate 1 standard error of the mean. A, B, C & D indicate the proportion of rightward directional judgements at the longest SOA (700 ms).

Figure 2 shows the proportion of rightward directional judgements at SOAs of −700, −400, −150, −90, −60, −30, −15, 15, 30, 60, 90, 150, 400 and 700 ms, with negative SOAs for stimuli going leftwards in space (e.g. leftwards according to an external spatial reference frame), and positive for stimuli going rightwards. It can be observed that participants seem to have reported directional judgements opposite to the actual stimulation direction when their fingers were crossed.

To investigate if the directional judgements in crossed conditions are indeed systematically inverted, a fitting algorithm was applied to the average group results. Both the data in uncrossed (Chi-square goodness of fit test χ^2^(11) = 3.760, p = .97) and crossed condition (χ^2^(11) = 4.872, p = .94) could be well described by a logistic curve (uncrossed: R^2^ = .992 and crossed: R^2^ = .974). Individual data also fitted well to a logistic curve in uncrossed condition (all R^2^>.83) and in most participants in crossed condition (R^2^>.69). In 2 out of 10 participants, the proportion of rightward directional judgements with crossed fingers could not be fitted to a logistic curve (R^2^ = .171 & R^2^ = .395) due to a high variability in judgements with short SOAs.

The total percentage of correct responses was different from chance level (50%) in both uncrossed condition (mean = 74.8% SE = 3.1%, T-test t(9) = 8.018 p<0.001) and crossed condition (mean = 31% SE = 3.8% T-test t(9) = −5.030 p = 0.001). Furthermore, the total percentage of correct responses was higher in uncrossed conditions than in crossed (Paired Sample T-tests t(9) = 8.751 p<0.001), showing a large crossing effect. The total percentage correct with crossed fingers was below chance level, again indicating that the participants reported perceiving the direction of the stimuli as opposite to what it really was.

To further investigate this reversal, we analysed the slope parameters of the functions per participant. The two participants whose responses with crossed fingers could not be fitted to a logistic curve were excluded from this analysis. Paired Sample T-tests showed a main effect of crossing the fingers on the slope parameters (on average 0.025 (SE = 0.005), corresponding to a change of 0.025 in proportion of rightward responses (scale 0–1, unitless) per millisecond SOA with uncrossed and −0.022 (SE = 0.010) in crossed fingers; p = 0.008, t(7) = 3.642), the signs indicating that the slopes are inverted in the crossed condition as can be seen in Figure 2. Single subject data showed that slopes of the functions were inverted in crossed as compared to uncrossed condition in 7 out of 8 participants, and a similar trend was observed for the 2 other participants, so in all but one participant the pattern of directional judgement was reversed when the fingers were crossed.

To check for differences in general performance between the two finger conditions, the absolute slope parameters of the functions (e.g. the numerical values without regards to their signs) were analysed per participant. The two participants whose responses with crossed fingers could not be fitted to a logistic curve were again excluded from this analysis. Paired Sample T-tests showed no effect of crossing the fingers on absolute slope values (on average 0.025 (SE = 0.005) with uncrossed and 0.031 (SE = 0.006) with crossed fingers; p = 0.355, t(7) = −0.991), indicating no difference in difficulty between crossed and uncrossed condition. (When the slope parameters of the two other subjects were included -though not well-fitted giving an indication- there was still no difference in absolute slope between crossed and uncrossed conditions (p = 0.677, t(9) = −0.526).)

As can be seen in figure 2, the proportion of rightward directional judgements in crossed condition at the longest SOA (700 ms) remained significantly different compared to uncrossed (On average: uncrossed (point B in fig. 2): 97% (SE = 1.9%) and crossed (point A): 74% (SE = 7.4%), Paired Sample t-test: t(9) = 3.411, p = 0.008, uncrossed (C): 2.8% (SE = 1.5%) and crossed (D): 24% (SE = 7.7%), Paired Sample t-test: t(9) = −2.835, p = 0.020). This difference remains significant when excluding the participant with “un-reversed” directional judgements (t(8) = 3.686, p = 0.006 and t(8) = −2.600, p = 0.032). In addition, directional judgement proportions stabilised at a SOA of around 300 ms in both crossed and uncrossed condition but the standard errors at higher SOAs were larger with crossed compared to uncrossed fingers, indicating a higher inter-subject variability.

### Reaction times

**Figure 3:**
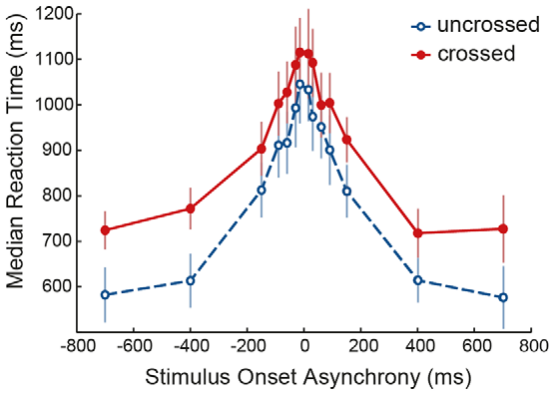
The mean of median reaction times (ms) at different SOAs. Mean median reaction times in trials with fingers uncrossed (blue, open circles, dashed line) and crossed (red, filled circles, continuous line) are depicted at different SOAs. Reaction times are defined as the time between the second tap and the response. Negative SOAs again indicate trials in which the direction of stimuli was to the left in space. Error bars indicate 1 standard error of the mean.

Figure 3 shows the median reaction times (RT), time between the second tap and the response, in trials with fingers uncrossed or crossed at different SOAs. A finger position (2)×SOA (7)×stimulus direction (2) repeated measures analysis showed a main effect of finger position (p = 0.002, F(1,9) = 19.341) with crossing the fingers leading to longer RTs (by on average 60–120 ms).

Furthermore there was a main effect of SOA (p<0.001, F(6) = 58.719) reflecting quicker responses with increasing SOA. This might indicate that the task was easier with longer SOAs, having more processing time for the first stimulus. If participants used a response strategy by reacting to the location of the first tap, we would expect that RT to the first stimulus does not change with increasing SOA. This, however, was not supported by the analyses of the RT calculated from the first tap. At least for the shorter SOAs, RTs with respect to the first tap still decreased with increasing SOA. Interestingly, for the longest two SOAs (400 & 700 ms), the RTs increased considerably (but not with respect to the second tap) (see Table 1). This pattern may be expected if a) the RTs with respect to the second tap at longer SOAs reached a point were participants could not respond much faster or b) participants based their response on the first tap and with longer SOAs had to wait for the second tap before they could respond. The latter is clearly not the case, as this would have resulted in a disappearance of the reaction time differences between crossed and uncrossed condition at longer SOAs. Therefore, it appears that participants in this experiment answered as quickly as they could, and based their responses not solely on the first tap, thus had to use both taps to form a perception of direction.

**Table 1:** RTs (ms) at different SOAs with respect to the first and second tap.

	RTs to 1th. tap (ms)		RTs to 2th. tap (ms)		SE (ms)	
SOA (ms)	uncrossed	crossed	uncrossed	crossed	uncrossed	crossed
15	1053	1113	1038	1098	81	82
30	1011	1104	981	1074	77	75
60	997	1067	937	1007	69	63
90	986	1094	896	1004	68	68
150	946	1062	796	912	59	56
400	1008	1139	608	739	52	43
700	1274	1414	574	715	60	52

Mean median reaction times (RTs) to the first and second tap at the different SOAs and 1 standard error of the mean. Reaction times to stimuli going leftwards and stimuli going rightwards are collapsed, since a finger position (2)×SOA (7)×stimulus direction (2) repeated measures analysis showed no main effect (or interaction) of stimulus direction.

## Discussion

In this study we investigated the perceived direction of movement of two tactile stimuli, applied successively to the crossed or uncrossed index and middle finger of one hand. The total percentage of correct responses with crossed fingers was significantly below chance level. Moreover, the slope of the psychometric function fitted to the proportion of rightward directional judgements at the different SOAs was inverted in crossed as compared to uncrossed condition. Thus, participants almost consistently reported perceiving the stimulus direction as opposite to what it actually was in the fingers crossed condition. This suggests that on average they did not integrate the crossed finger position when making their directional judgements. Therefore the results are in agreement with the idea that, by default, the processing of tactile stimuli applied to the fingers assumes a prototypical positioning of body parts [4]. However, in contrast to what has been generally found for tactile perception with crossed hands (see for instance [4], [5], [7], [8], [14], [22], [28]), performance did not change to incorporate the unusual finger postures for SOAs as long as 700 ms. This indicates that time between the two stimuli alone was not sufficient to allow for the crossed posture information to be integrated with the tactile information. Several aspects may be relevant to explain these results, such as haptic experience, incomplete remapping and differential weighting of reference frames, and will be discussed below in relation to the results of previous studies.

### Comparison with other crossed finger experiments

Our results are in agreement with those of Sekine and Mogi [25], who showed reversed directional judgements when crossing the fingers, suggesting incomplete integration of the crossed posture. In addition to their findings, we show that this reversal does not subside with longer SOAs. As was outlined in the introduction, not every study investigating tactile perception with crossed fingers finds this lack of integration of the crossed posture. For instance, Craig [24] asked participants to judge the direction of apparent motion of two successive tactile stimuli to crossed fingers and observed that participants accurately incorporated the crossed fingers posture. However, as the participants could see their crossed fingers, the integration of the posture might be based on visual information. Indeed, several authors have suggested that visual information about the crossed posture might facilitate coding in an external spatial reference frame [5]–[7], [29], [30]. As discussed by Cadieux [7], several manipulations of the vision of the limbs influence crossing effects, for example crossing the hands behind the back [31] or manipulating the visual information about the crossing of the hands with (un)crossed rubber hands [6]. Furthermore, while sighted individuals showed impaired temporal order judgements with crossed hands at short SOAs, congenitally blind did not show this effect [29]. Therefore, visual input informative of body part posture might partly explain the difference between Craig's [24] and our data. A second factor that might explain the difference in results is stimulus complexity. In contrast to the simple tactile stimuli used in the current study and by Sekine and Mogi [25], Craig used a rather complex tactile display. Each stimulus consisted of a local apparent motion pattern generated by an array of 144 tactile stimuli, and stimulus presentation lasted 65 ms. Both the complexity and the longer stimulus duration might have allowed participants to build a more robust percept of the tactile stimulus, as compared to two short tactile taps. Similarly, a recent study by Kuroki et al. [32], used various SOAs ranging from 0 to 180 ms and tactile stimuli consisting of vibrating pins, with a stimulus duration of 200 ms. Additionally, participants were allowed to keep their eyes open. This study found that a tactile motion after effect, induced by repetitively applying the two stimuli in the same order, and the perceived direction of the apparent motion (subsidiary experiment) was determined by the direction of the stimuli in space. Thus in their experiment, the posture was included in the processing of tactile stimuli to the fingertips. Again, the combination of visual information about the crossed posture, and the long stimulus duration may have allowed participants to process the information more substantially.

In terms of posture and range of SOAs our experiment resembles that of Heed et al. ([25], experiment 3). As in the current study, they found an incomplete integration of the crossed posture and no re-reversal of the of the temporal order judgements at longer SOAs. However, while we found mostly no integration of the crossed posture, as was shown by an inverted S-shaped curve of the responses with the crossed as compared to the uncrossed posture, Heed et al. reported an almost flat, uninverted, response curve. The flatness may reflect a large variance between subjects, as is mentioned by Heed et al. in their results section. The fact that it is uninverted reveals substantial integration of the crossed finger posture. Possibly, the difference in between-subject variance is caused by the dissimilarity in both the task and response methods. Heed et al. asked participants to perform a finger identification (TOJ) task. Fingers have non-spatial identities (e.g. index finger or little finger) and tasks asking for finger identification therefore might not require incorporation of postural information. This task requirement in itself could thus have yielded no interference of the crossed posture in the experiment of Heed et al. However, participants were allowed to keep their eyes open, and although they were instructed to look at a fixation cross, the hands were not shielded from view. As discussed in the previous paragraph, visual input may facilitate integration of the crossed posture. Furthermore, responses were given using a foot pedal. In contrast to for instance verbal responses, this requires spatial target-response mapping [33]. The spatial target-response mapping and the availability of vision may have caused some participants to adopt a more external reference frame. Combined, these factors may have caused larger differences between subjects. Furthermore, inspection of their figure 3A shows that the fingers were closer to each other in space in the fingers crossed as compared to the fingers uncrossed condition. This may have caused an increase in the error rate (making the response curves flatter), since tactile stimuli can more easily be mislocated to the wrong finger when the fingers are closer to each other [3], [34].

Indeed, both task and response requirements have been found to influence crossing effects. Sekine and Mogi [24] found no influence of crossing the fingers on judgements of which finger was tapped last (index or middle), but did see a considerable decrease in performance on a directional judgement task. However, prior to the experiment, participants practised the finger identification but not the directional judgement task. Still, a similar result was reported by Roberts & Humphreys [27], who found crossing effects in temporal order judgements only when asking to report spatial attributes. Additionally, Gallace et al. [35] compared the influence of different response methods on crossing effects. In their experiment, the influence of a distractor to one hand on a tactile target stimulus on the other hand was determined in spatial coordinates when subjects had to respond using a foot pedal, even when the task at hand did not require doing so (e.g. finger identity judgements), but less so when they had to respond verbally. When the target finger had to be lifted the response pattern was no longer spatially defined. The authors concluded that response demands, to a large extent, determine the amount of remapping of tactile information to an external frame of reference.

### Differences in remapping between hands and fingers

It has been debated whether fingers and hands are represented differently. For instance, in a study by Haggard et al. [36] participants had to indicate which hand or which finger had been touched in two different postures. While hand identification performance declined when the fingers of the two hands were interleaved, finger identification was not influenced by this posture. Haggard et al. concluded that fingers are represented in a somatotopic space, whereas hands are represented in an egocentric external space. However, as Riemer et al. [37] pointed out, in these conditions there may be differences in interfering influences of crossing the hands and fingers together compared to crossing only fingers or hands. Indeed, crossing effects on a temporal order task are larger when crossing both the hands and the fingers as compared to either crossing fingers or hands, suggesting an interference between crossing hands and fingers [26]. In the current experiment we tried to minimise this problem by crossing the fingers of one hand. Our results show that when processing tactile stimuli to the fingertips, unusual postures are not necessarily integrated. When crossing hands however, an incorporation of the crossed posture with longer SOAs is generally observed [4], [5], [7]–[9], [15], [26].

Differences in the influence of crossing the hands and fingers may be explained by the lack of haptic explorative experience with crossed fingers [22]. The hands move constantly, and they often cross over each other. However, crossing your fingers is not an everyday situation. Indeed, Benedetti [23] showed that only after several months of training crossed fingers with concurrent multimodal input (tactile, visual and proprioceptive) by taping them together for several hours a day, the perceptual reversal in space declined. The crossed posture in our experiment can only be obtained by actively forcing the fingers in that position, something you would not likely do in haptic explorative behaviour. It could therefore be suggested that the lack of integration of the unusual finger postures with long SOAs observed in our experiment is due to the haptic inexperience with the crossed posture. Thus, instead of concluding that tactile stimuli to the fingers are processed according to one or the other reference frame, we suggest (in line with [7], see also [38]) that differential weighing between different representations occurs, depending on the task at hand, the availability of different sensory input and the prior experience we have with tactile processing in a certain posture.

### Incomplete remapping

The integration of the crossed posture is not an ‘all or nothing’ process. Indeed, in our study the “reversal effect” (spatial reversal in response pattern) was not complete, as can be revealed by the lack of an exact mirroring of the proportion of responses right- or leftwards in the uncrossed posture (about 70% instead of about 95%). This difference cannot be explained by an increase in task difficulty with crossed fingers, as the slopes of the curves did not differ between both conditions. Therefore we suggest that the crossed posture was processed to some extent. The integration of a posture has been explained as a translation or transference between different reference frames. Tactile information is processed first according to a somatotopic reference frame. When tactile information is used for a spatial task, a translation from a somatotopic to an external spatial reference frame would be required to achieve the correct answer (remapping of touch; see for instance [39]). Several studies have suggested that somatotopic and external spatial representations are formed in parallel [40], [41] and differences in perception are a consequence of the weight attributed to each during the integration [7] (similar to for instance tactile-auditory [42], tactile-visual [43] and haptic-visual integration [44]). As discussed above, these weights could depend on task requirements [25], [27], response methods [35] differences in stimulus characteristics (compare the current results with [24]), the availability of visual information [7], strategy [7] and experience in processing tactile information in a certain posture. In Benedetti's studies the somatotopic representation dominated and the postural position was not incorporated [20], [22], [45]. In our experiment the postural information was incorporated in some trials, but it is still considerably less as compared to tactile perception with crossed hands. Moreover, long SOAs did not allow for the incorporation of the crossed posture (see also [22]), as is seen in experiments with crossed hands [4], [5], [7]–[9], [15], [26]. Therefore, the current experiment suggests that the localization of stimuli according to a somatotopic reference frame and the integration of postural information are two separate processes that apply differently to the hands and fingers.

### Conclusion

To conclude, this study demonstrates that the somatotopic representation of tactile stimuli is not necessarily remapped to a spatial reference frame to include the crossed posture during the construction of a stable percept. In this experiment, participants almost consistently reported perceiving the stimulus direction of two tactile stimuli presented to crossed fingers as opposite to what it was. This suggests that during the construction of the percept, the somatotopic representation was weighted heavier than the postural information, rendering responses mostly based on somatotopic processing. Our results therefore support the suggestion that tactile processing involves a weighted integration of somatotopically-based representations with postural information. While for hands, atypical postures are usually included in the constructed percept, particularly when sufficient time is allowed, the representation of fingers can remain mostly somatotopically based. This suggests that the localization of stimuli in a somatotopic reference and the integration of postural information are two separate processes that apply differently to the hands and fingers.
